# *Athyrium haleakalae* (Athyriaceae), a new rheophytic fern species from East Maui, Hawaiian Islands: with notes on its distribution, ecology, and conservation status

**DOI:** 10.3897/phytokeys.76.11637

**Published:** 2017-01-19

**Authors:** Kenneth R. Wood, Warren L. Wagner

**Affiliations:** 1 National Tropical Botanical Garden, 3530 Papalina Road, Kalāheo, HI 96741, USA; 2 Department of Botany, Smithsonian Institution, PO Box 37012, Washington, DC 20013-7012, USA

**Keywords:** Athyriaceae, Athyrium, new species, rheophyte, Hawaiian Islands, East Maui endemic, Critically Endangered

## Abstract

*Athyrium haleakalae* K.R. Wood & W.L. Wagner (Athyriaceae), a small lithophytic fern from East Maui, Hawaiian Islands, is described and illustrated. Notes on its distribution, ecology, and conservation status are also presented. The new species appears to be an obligate rheophyte, preferring sites of fast moving water along concave walls of streams and waterfalls. *Athyrium haleakalae* differs from the only other known Hawaiian *Athyrium*, *Athyrium microphyllum* (Sm.) Alston, in having rhizomes 1–3 cm long and lanceolate blades 1- to 2-pinnate-pinnatifid, 3–8(–11) × 1–3(–4) cm, as compared to *Athyrium microphyllum* having rhizomes (10–)15–30 cm long and ovate to ovate-triangular blades 3-pinnate-pinnatifid to 4-pinnate, 30–82 × 20–50 cm.

## Introduction

*Athyrium* Roth, in the family Athyriaceae Alston, is a genus composed of ca. 230 species of terrestrial or epilithic plants with mostly erect or occasionally creeping or ascending rhizomes. Primary centers of *Athyrium* diversity are found in the Sino-Himalayan region where ca. 91 species are recorded from Southwest China, Sichuan Basin, Tibet-Yunnan Plateau and Nepal (Kramer and Green 1990, Fraser-Jenkins 1997, Wang 1999, Mabberley 2008, Liu et al. 2009) and with secondary centers of diversity in the Western Pacific islands where ca. 54 species are known from the Japanese Archipelago, the Ryukyu Islands, Taiwan, and the Philippines (Salgado 1990, Kato 1995, Liu et al. 2009).

Concepts in the classification of *Athyrium* continue to change and evolve with recent molecular phylogenetic studies (Smith et al. 2006, Christenhusz et al. 2011, Rothfels et al. 2012, Sundue and Rothfels 2014, PPG I 2016). Smith et al. (2006) published a revised fern classification based on both morphological and molecular evidence placing *Athyrium* into Woodsiaceae (Diels) Herter, yet state that its placement was tentative and in need of a more refined analysis. Subsequently Christenhusz et al. (2011) placed *Athyrium* into Athyriaceae along with four other genera, namely *Anisocampium* C. Presl, *Cornopteris* Nakai, *Deparia* Hooker & Grev., and *Diplazium* Sw. Furthermore, they report the need for continued research, referring to the monophyly of *Athyrium* and *Diplazium*. In 2016 the Pteridophyte Phylogeny Group (PPG) published the most current understanding of lycophyte and fern phylogeny and in their community-derived classification they limit Athyriaceae to three genera, namely *Athyrium*, *Deparia*, and *Diplazium*, with an estimated 650 species.

There are nine other athyrioid fern species endemic to the Hawaiian Islands, namely *Athyrium microphyllum* (Sm.) Alston, *Deparia cataracticola* M. Kato, *Deparia fenzliana* (Luerss.) M. Kato, *Deparia kaalaana* (Copel.) M. Kato, *Deparia marginalis* (Hillebr.) M. Kato, *Deparia prolifera* (Kaulf.) Hook. & Grev., *Diplazium arnottii* Brack., *Diplazium molokaiense* W.J. Rob., and *Diplazium sandwichianum* (C. Presl) Diels (Palmer 2003, Vernon and Ranker 2013). This recent discovery and present publication of *Athyrium haleakalae* K.R. Wood & W.L. Wagner brings the total number of Hawaiian Athyriaceae to ten, and represents the second *Athyrium* species in the archipelago.

## Methods

All measurements were taken from dried herbarium specimens and field notes and are presented in the descriptions as follows: length × width, followed by units of measurements (mm or cm). The authors have examined all specimens cited. The extent of occurrence and area of occupancy for *Athyrium haleakalae* was calculated by using ArcMap 10.2 in relation to coordinates recorded while collecting herbarium specimens or making field observations

## Taxonomic treatment

### Athyrium haleakalae

Taxon classificationPlantaePolypodialesAthyriaceae

K.R. Wood & W.L. Wagner sp. nov.

urn:lsid:ipni.org:names:77159814-1

Figs 1 , 4A

#### Diagnosis.

*Athyrium haleakalae* differs from the only previously known Hawaiian *Athyrium*, *Athyrium microphyllum*, in having rhizomes 1–3 cm long and lanceolate blades 1- to 2-pinnate-pinnatifid, 3–8(–11) × 1–3(–4) cm, as compared to *Athyrium microphyllum* with rhizomes (10–)15–30 cm long and ovate to ovate-triangular blades 3-pinnate-pinnatifid to 4-pinnate, 30–82 × 20–50 cm.

**Figure 1.**
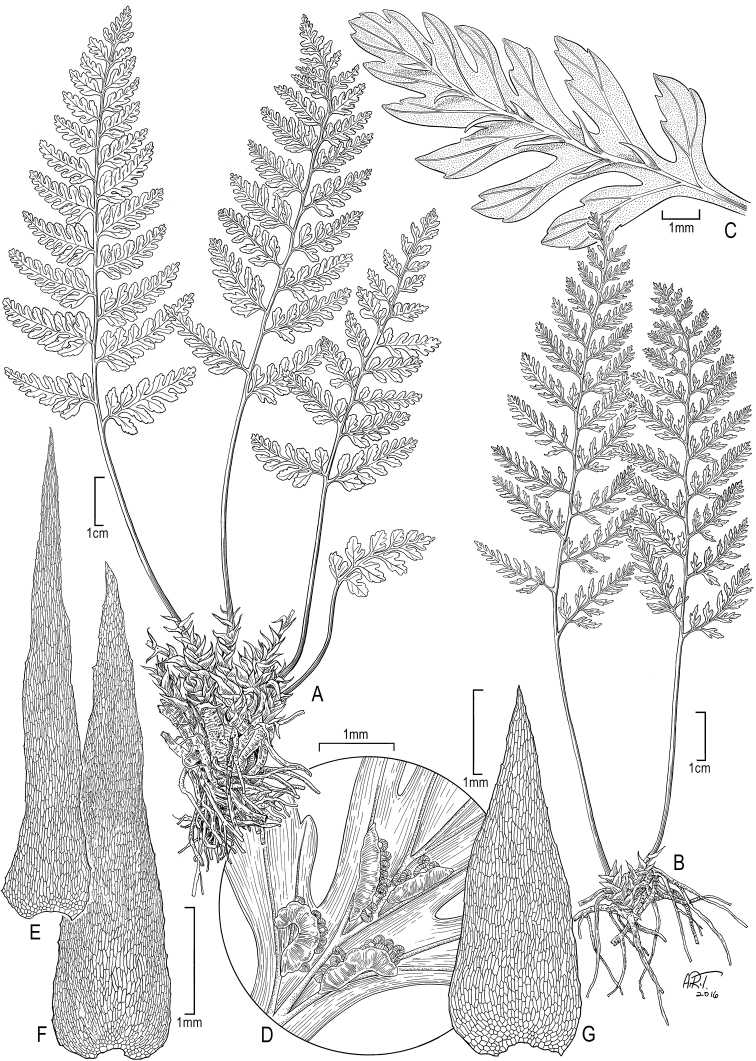
*Athyrium haleakalae* K.R. Wood & W.L. Wagner. **A–B** habit **C** detail of adaxial pinnule showing venation and fleshy spines **D** detail of abaxial pinnule showing range of sori shapes **E–F** lower stipe scales **G** rhizome scale. **A–G** from *Perlman et al. 23964* (BISH, PTBG, UC, US) (Illustration by Alice Tangerini).

#### Type.

United States of America. Hawaiian Islands, East Maui: Ko‘olau Forest Reserve, west fork of Helele‘ike‘oha Stream, 20°45'14.58"N; 156°5'23.496"W, 1247 m elev., 28 May 2014, *Perlman et al. 23964*, (holotype: PTBG-070914; isotypes: BISH, UC, US).

#### Description.

*Lithophytic ferns*. *Rhizomes* slender, erect to suberect, unbranched, radial, dark brown, 1–3 × 0.5–1.0 cm, closely set with roots and persistent, densely clothed by old stipe bases; scales covering rhizome tips, stramineous to dark brown, 2–4 × 1.0–1.5 mm, lanceate, margins entire, attenuate toward apex. *Fronds* 3–7 per rhizome; *stipes* medium brown, 20–50(–70) × 0.3–0.7 mm, swollen bases proximally thickened to 1 mm, well clothed with stramineous to dark brown basal scales 3.0–4.5 × 0.5–1.0 mm, sparser distally, thinning to glabrous; *blades* medium green, 1- to 2-pinnate-pinnatifid, 3–8(–11) × 1–3(–4) cm, lanceolate, *rachises* medium green to dark brown, glabrous, apex pinnatifid, acute, lobed ½ toward costae, *pinnae* 10–12 pair, lanceolate, alternate, petioled 1–2 mm, sessile near apex, fleshy spines 0.3–1.0 mm long on adaxial surface at bases of costae and costules, basal pinnae spaced 2–5(–8) mm, distal pinnae more closely spaced, not overlapping, lowest pinnae slightly reduced, second lowest pair usually largest, 0.7–2.0(–2.5) × 0.3–0.5(–0.8) cm, *pinnules*, lower with 6–8 pair, reduced distally, ovate to lanceolate, near alternate, serrate to lobed, veins 2–4 pairs in basal lobe, fewer pairs distally. *Sori* short linear, oblong, or J-shaped, 1.0–1.4 mm long, along acroscopic base of veinlets, 1(–2) per ultimate segment, *indusia* tan or brown, same shape as sori, entire, persistent.

#### Etymology.

The new species is named after Haleakalā, East Maui, a massive, dormant shield volcano (3,057 m tall) and the only known location of *Athyrium haleakalae*.

#### Specimens examined.

**United States. Hawaiian Islands, East Maui**: Hana Forest Reserve, Mokulehua drainage basin, *Metrosideros*-*Cheirodendron*-*Dicranopteris* montane wet forest, dissected by riparian vegetation, 1195 m elev., 21 Aug 2013, *Wood & Oppenheimer 15624* (BISH, PTBG, US); *loc. cit.*, 1161 m elev., 21 Aug 2013, *Oppenheimer et al. H81332* (BISH, PTBG); Hana Forest Reserve, Kawakoe headwaters, 1183 m elev., 22 Aug 2013, *Wood et al. 15637* (PTBG); *loc. cit.*, 1164 m elev., 22 Aug 2013, *Wood et al. 15639* (PTBG, UC); Ko‘olau Forest Reserve, west fork of Helele‘ike‘oha Stream, 1326 m elev., 28 May 2014, *Oppenheimer et al. H51415* (NY, PTBG); *loc. cit.*, 1367 m elev., 28 May 2014, *Oppenheimer et al. H51418* (MO, PTBG); *loc. cit.*, 1204 m elev., 29 May 2014, *Oppenheimer et al. H51426* (PTBG, UC); Haleakalā National Park, Kīpahulu Valley, south of Palikea Camp, 1280 m elev., 28 Aug 2014, *Welton et al. 2359* (HALE).

#### Key to *Athyrium* in the Hawaiian Islands

**Table:** 

1	Plants lithophytic; blades lanceolate 1- to 2-pinnate-pinnatifid, 3–8(–11) × 1–3(–4) cm	***Athyrium haleakalae***
–	Plants mostly terrestrial; blades ovate to ovate-triangular 3-pinnate-pinnatifid to 4-pinnate, 30–82 × 20–50 cm	***Athyrium microphyllum***

#### Distribution and ecology.

*Athyrium haleakalae* has only been documented on the mountain of Haleakalā, East Maui, the third highest prominence in the Hawaiian archipelago at 3,057 m. The volcanic island of Maui is ca. 1.2 million years old (Price and Elliott-Fisk 2004) and has an area of ca. 1,884 km^2^. Hawaiian flowering plants on Maui include 518 plant taxa, with 422 endemic and 99 of those being single-island endemics (Sakai et al. 2002). Estimates on the number of endemic fern and lycophyte taxa on Maui vary only slightly (Palmer 2003, Vernon and Ranker 2013) and the present authors estimate there are 115, including five single-island endemics. *Athyrium haleakalae* now represents the sixth single-island endemic fern or lycophyte taxon found on Maui. It is the tenth endemic athyrioid species in the Hawaiian Islands, and the second in that group that is restricted to a single island, the other being *Deparia cataracticola* M. Kato, of Kaua‘i.

**Figure 2.**
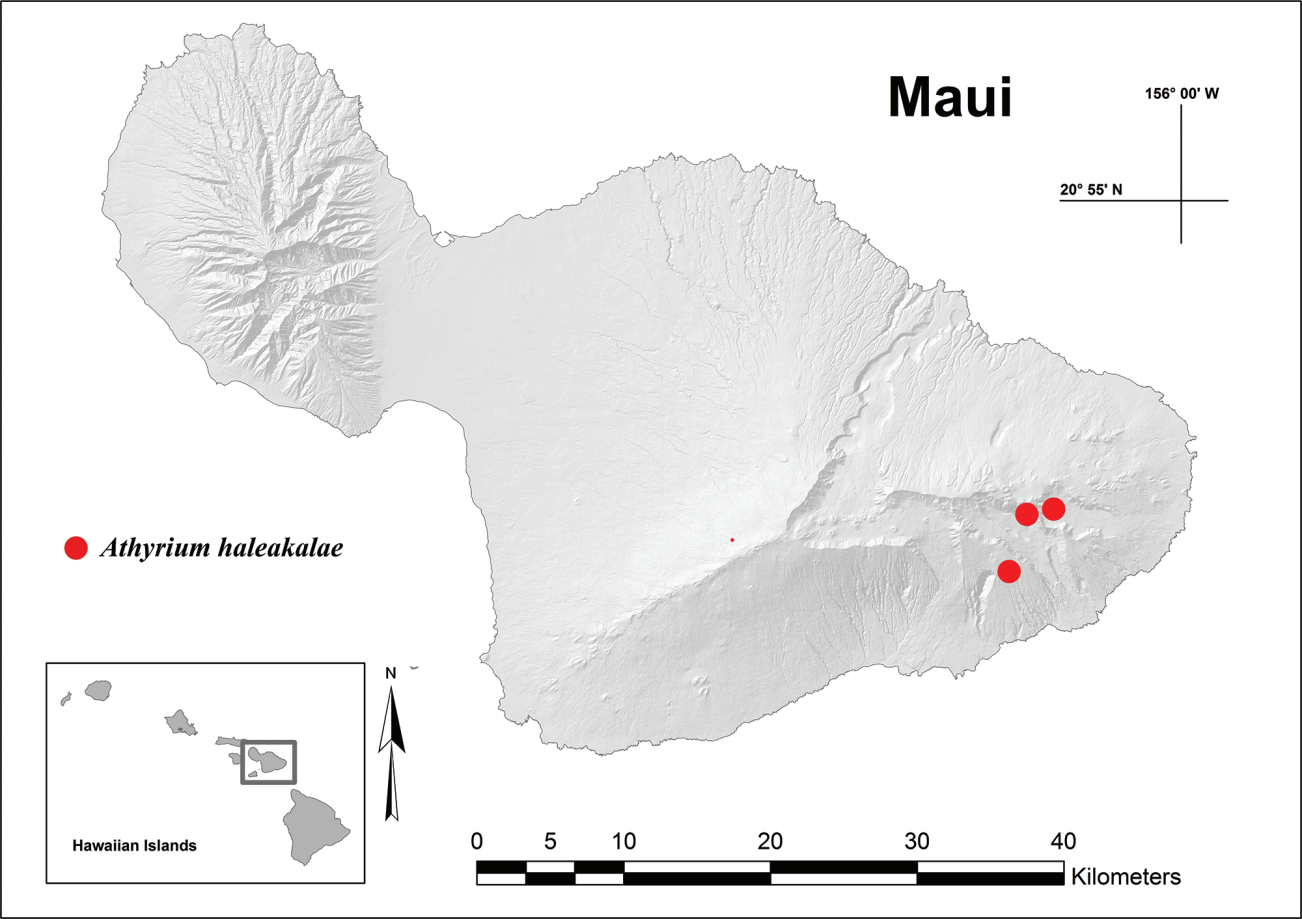
Map showing known distribution of *Athyrium haleakalae*, East Maui, HI, with upper right red dot indicating colonies in the headwaters of Kawakoe and Mokulehua, upper left in Helele‘ike‘oha, and lower red in Kīpahulu, near Palikea.

Since its discovery in August of 2013 ca. 300 plants of *Athyrium haleakalae* have been observed in several headwater drainage systems of East Maui, namely Mokulehua and Kawakoe in the Hana Forest Reserve, Helele‘ike‘oha in the Koolau Forest Reserve, and Kīpahulu, near Palikea in Haleakalā National Park (Figure 2). Perhaps the combination of its small size, remoteness of preferred habitat, and the extreme physical geography of its surroundings can explain why *Athyrium haleakalae* has been overlooked to date. Modern access by helicopter and careful floristic inventories around large waterfalls and rugged plunge pools have led to its recent discovery by botanists of the National Tropical Botanical Garden
(NTBG), the Maui Nui Plant Extinction Prevention Program (PEPP), and Haleakalā National Park. It is believed that the extent of occurrence for *Athyrium haleakalae* may be greater than the four drainages reported here, and further research into similar habitats along adjacent drainage basins could lead to the discovery of additional colonies.

**Figure 3.**
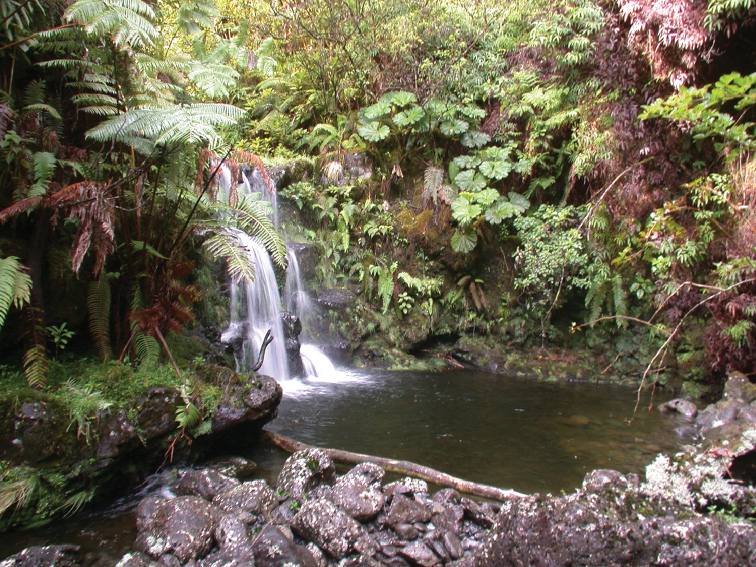
Typical habitat of *Athyrium haleakalae* around stream plunge pools, Hana Forest Reserve, East Maui, HI. Photo by K.R. Wood, 21 Aug 2013.

**Figure 4.**
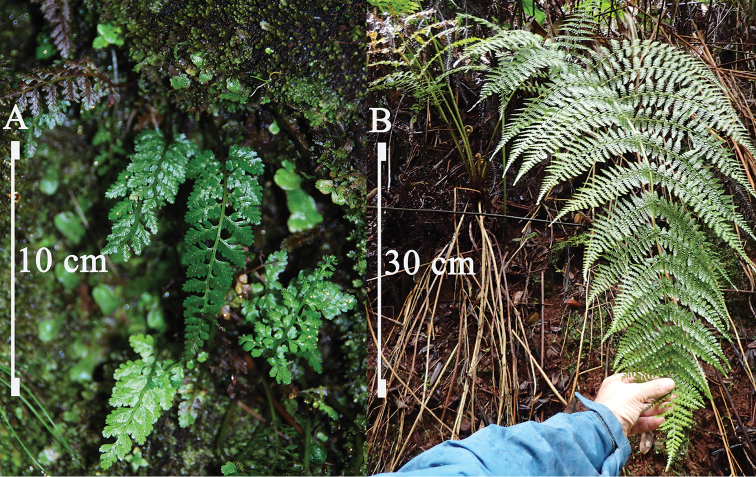
**A** Mature plants of *Athyrium haleakalae*, showing habitat preference along concave hollow of stream, Hana Forest Reserve, East Maui, HI (22 Aug 2013, *Wood & Oppenheimer 15639*) **B** Mature plant of *Athyrium microphyllum*,﻿ showing terrestrial habitat preference, erect rhizome, and large size, Mohihi, Kaua‘i, HI (18 Dec 2014, *Wood & Flynn et al. 16175*). Photos by K.R. Wood.

The current distribution of *Athyrium haleakalae* has an elevational range of 1,161–1,326 m. The dominant plant community of those regions is a *Metrosideros* Banks ex Gaertn. (Myrtaceae)-*Cheirodendron* Nutt. ex Seem. (Araliaceae) montane wet forest. Large colonies of matting ferns such as *Dicranopteris linearis* (Brum. f.) Underw. and *Diplopterygium pinnatum* (Kunze) Nakai (both Gleicheniaceae) are associated with these forests, being especially expansive near forest borders where steep slopes drop down to deep dissecting streams. Observations to date indicate that *Athyrium haleakalae* is an obligate rheophyte which prefers concave moss-matted basalt walls along the waterline of perennial streams, forming colonies over wet basalt rock faces especially under and around the ledges of waterfalls and hollows of large plunge pools (Figures 3, 4A). These stream sites average ca. 10–15 m broad and have exposed basalt bedrock and large strewn boulders. Associated ferns occurring with *Athyrium haleakalae* include *Athyrium microphyllum*, *Cyclosorus sandwicensis* (Brack.) Copel. (Thelypteridaceae), *Selaginella arbuscula* (Kaulf.) Spring (Selaginellaceae), and *Hymenasplenium unilaterale* (Lam.) Hayata and *Vandenboschia davallioides* (Gaudich.) Copel. (both Hymenophyllaceae). Significantly, *Athyrium haleakalae* grows in association with one of the rarest Hawaiian endemic rheophytes, *Cyclosorus boydiae* (D.C. Eaton) W.H. Wagner.

Adjacent riparian angiosperm vegetation, usually outside the rheophyte zone, includes *Broussaisia arguta* Gaudich. (Hydrangeaceae), numerous species of *Clermontia* Gaudich. and *Cyanea* Gaudich. (both Campanulaceae), several species of *Cyrtandra* J.R. Forst. & G. Forst. (Gesneriaceae), *Deschampsia nubigena* Hillebr. (Poaceae), *Dubautia plantaginea* Gaudich. and *Dubautia scabra* (DC.) D.D. Keck (both Asteraceae),﻿ *Gunnera petaloïdea* Gaudich. (Gunneraceae), *Kadua affinis* DC. and *Kadua axillaris* (Wawra) W. L. Wagner & Lorence (both Rubiaceae), *Labordia venosa* Sherff (Loganiaceae), *Machaerina angustifolia* (Gaudich.) T. Koyama (Cyperaceae), *Melicope clusiifolia* (A. Gray) T.G. Hartley & B.C. Stone and *Melicope molokaiensis* (Hillebr.) T.G. Hartley & B.C. Stone (both Rutaceae), *Myrsine sandwicensis* A. DC. (Primulaceae), *Nertera granadensis* (Mutis) Druce (Rubiaceae), *Phyllostegia ambigua* (A. Gray) Hillebr. (Lamiaceae), *Polyscias oahuensis* (A. Gray) Lowry & G.M. Plunkett (Araliaceae), *Scaevola chamissoniana* Gaudich. (Goodeniaceae), ﻿and *Vaccinium dentatum* Sm. (Ericaceae).

Pigs (*Sus scrofa* L.), landslides, and invasive weeds such as *Ageratina adenophora* (Spreng.) R.M. King & H. Rob. (Asteraceae), *Axonopus fissifolius* (Raddi) Kuhlm. and *Paspalum urvillei* Steud. (both Poaceae), *Juncus planifolius* R. Br. (Juncaceae), *Hedychium gardnerianum* Ker Gawl. (Zingiberaceae), and *Tibouchina herbacea* (DC.) Cogn. (Melastomataceae) threaten the immediate habitat of *Athyrium haleakalae*.

#### Conservation status.

*IUCN Red List Category.* When evaluating the conservation status of *Athyrium haleakalae* utilizing the World Conservation Union (IUCN) criteria for endangerment (IUCN 2001), *Athyrium haleakalae* falls into the Critically Endangered (CR) category, which designates this species as facing the highest risk of extinction in the wild. Our formal evaluation can be summarized by the following IUCN hierarchical alphanumeric coding system of criteria and subcriteria: CR B1ab(i,ii,iii,v)+2ab(i,ii,iii,v), which reflects a wild population of ca. 300 individuals, an Extent of Occurrence (EOO) of 4.3 km^2^, and an Area of Occupancy (AOO) of less than 1 km^2^. Ecosystem trends on the mountain of Haleakalā also indicate that *Athyrium haleakalae* is subject to an inferred decline in its area of occupancy, in addition to a decline in the extent and quality of its habitat and number of mature individuals. It should be noted that *Athyrium haleakalae* is currently being cultivated by the Hawai‘i State Division of Forestry and Wildlife at their Olinda Rare Plant Facility on East Maui.

## Discussion

*Athyrium microphyllum*, previously thought to be the only member of the genus in the Hawaiian Islands, is widely distributed and endemic on all the major high islands, ranging from 500 to 2,320 m (Figure 4B). Hawaiians called this species ‘ākōlea and it is commonly found in native forest understory where it can occasionally become a co-dominant in healthy forests, especially just above the riparian edges of both mesic and wet forest habitats. *Athyrium microphyllum* is almost always terrestrial, averaging 50–100 cm tall, and having strait narrow rhizomes of up to 30 cm. Although *Athyrium microphyllum* is occasionally lithophytic around streams, its large form eventually gets dislodged from the walls that enclose fast moving waters especially during flash floods.

In great contrast, *Athyrium haleakalae* is quite unique and has adapted itself to a very specific habitat with the ability to withstand flooding torrents that cascade through the deeply carved drainages of East Maui. With its tenacious rhizomes 1–3 cm long, small recumbent lanceolate blades 1- to 2-pinnate-pinnatifid, 3–8(–11) × 1–3(–4) cm, and stipe scales of up to 4.5 mm long, *Athyrium haleakalae* can easily be distinguished from *Athyrium microphyllum* which has rhizomes (10–)15–30 cm long, ovate to ovate-triangular blades 3-pinnate-pinnatifid to 4-pinnate, 30–82 × 20–50 cm, and stipe scales of up to 15 mm long (Figures 1, 4A,B).

Currently there are no extra-Hawaiian species of athyrioid ferns naturally occurring in the Hawaiian archipelago, although there are two historical introductions that have naturalized, namely *Deparia petersenii* (Kunze) M. Kato and *Diplazium esculentum* (Retz.) Sw. In our review, no previously described *Athyrium* was comparable to *Athyrium haleakalae*, and with hopes for its conservation and habitat protection we report this newly discovered, critically endangered, narrow endemic rheophyte as the latest addition to the pteridophyte flora of the Hawaiian Islands.

## Supplementary Material

XML Treatment for Athyrium haleakalae
